# Supplementary material for: Converse flexoelectric two-dimensional MoS2 actuator
Source: Nat Commun. 2026 Feb 9;17:2519. doi: 10.1038/s41467-026-69271-w (PMC12996291; doi:10.1038/s41467-026-69271-w)
Supplement: Supplementary file 1 — Supplementary Information [file 41467_2026_69271_MOESM1_ESM.pdf]

Supplementary Information

Converse Flexoelectric Two-dimensional MoS_2 Actuator

Yeageun Lee[1,2,†], Hyung Jong Bae[1,†], Md Farhadul Haque[1], Keon-Hee Lim[1], Jin Myung Kim[2,3], Weilin Guan[2], and SungWoo Nam[2,4,*]

[1]Department of Mechanical Science and Engineering, University of Illinois at Urbana Champaign, Urbana, IL 61801, USA

[2]Department of Mechanical and Aerospace Engineering, University of California, Irvine, Irvine, CA 92697, USA

[3]Department of Materials Science and Engineering, University of Illinois at Urbana-Champaign, Urbana, IL 61801, USA

[4]Department of Materials Science and Engineering, University of California, Irvine, Irvine, CA 92697, USA

*Corresponding Author (E-mail: sungwoo.nam@uci.edu)

†These authors contributed equally to this work.

Supplementary Note

(1) Calculation of Q factor for including damping in the simulation

In order to accurately calculate the harmonic displacement, we first estimated the Q factor from experimental data and apply it to the simulation. Figure S9 shows the frequency-displacement plot near the 5th resonant frequency. The Q factor was calculated by dividing resonant frequency by the width of the peak at its half height (Δf), which is 20,700 Hz / 690 Hz = 30.

(2) Estimation of harmonic displacement of mono-, bi-, and tri-layer MoS_2 actuators

While we were able to simulate up to 0.20 mm for monolayer MoS_2 actuators due to aspect ratio limitations, the thicker MoS_2 layer enables us to simulate the resonant frequencies and harmonic displacement of the longer beam. Figure S7a and b show the 5th harmonic frequency and displacement of mono-, bi-, and tri-layer MoS_2 actuators with different lengths. As with the monolayer case, bi- and tri-layer devices also show quadratically decreasing resonant frequency and quadratically increasing harmonic displacement as the beam length increases. In addition, bilayer MoS_2 actuator shows lower harmonic displacement compared to the monolayer MoS_2, agreeing well with our experimental results.

(3) Estimation of actuation from piezoelectric effect, 1133-converse flexoelectric stress, electromagnetic stress, and Joule heating

Using the 200 μm-long model with monolayer MoS_2, we also calculated the contributions of other possible mechanisms to the 5th harmonic displacement, including the piezoelectric effect, 1133-converse flexoelectric stress, electromagnetic stress, and Joule heating. In case of the 1133-converse flexoelectric stress, we applied 1.41 nC/m for μ_{1133}, the same value as the estimated μ_{1111}

value. Table S1 summarizes the parameters used in each simulation, and Figure S8 illustrates the device actuation resulting from each effect. The maximum displacements obtained from the 1111- and 1133-converse flexoelectric effects, piezoelectric effect, and electromagnetic stress are 1.64, 0.014, 0.30, and 0.29 nm, respectively. The displacement resulting from Joule heating is more than 10 orders of magnitude smaller than those from the other effects and can therefore be neglected. These results strongly support that the 1111-converse flexoelectric effect is the dominant mechanism driving device actuation, unless monolayer MoS_2 exhibits a μ_{1133} value that is significantly higher than μ_{1111}. Additionally, when these other effects are taken into consideration, μ_{1111} is estimated to be in the range of 1.41 ± 0.52 nC/m, under the assumption that $\mu_{1111} = \mu_{1133}$.

Table S1. Simulation parameters

Module	Parameter	Value
**Solid Mechanics**	Young's modulus, Au	70 GPa
	Poisson's ratio, Au	0.44
	Young's modulus, Ag	83 GPa
	Poisson's ratio, Ag	0.37
	Young's modulus, Parylene-C	2.75 GPa
	Poisson's ratio, Parylene-C	0.4
	Elastic modulus, C_{11}, MoS_2	128.4 GPa [1]
	Elastic modulus, C_{22}, MoS_2	128.4 GPa [1]
	Elastic modulus, C_{12}, MoS_2	32.6 GPa [1]
	Elastic modulus, C_{33}, MoS_2	38.3 GPa [2]
	Elastic modulus, C_{44}, MoS_2	26.5 GPa [2]
	Elastic modulus, C_{55}, MoS_2	26.5 GPa [2]
	Elastic modulus, C_{66}, MoS_2	47.9 GPa<br>$((C_{11}-C_{12})/2)$
	Flexoelectric coefficient, μ_{1111}, MoS_2	1.41 nC/m
	Flexoelectric coefficient, μ_{1133}, MoS_2	1.41 nC/m
	Piezoelectric coefficient, e_{11}, MoS_2	0.5 C/m^2 [3]
	Isotropic structural loss factor (1/Q factor)	1/30
**Electrostatics & Electric Currents**	Terminal voltage, top electrode (Au)	20 V
	Terminal voltage, bottom electrode (Ag)	0 V
	Relative permittivity, Parylene-C	3
	Relative permittivity, MoS_2	4 [4]
**Heat Transfer in Solids**	Thermal conductivity, Au	317 W/m·K
	Density, Au	19300 kg/m^3
	Heat capacity, Au	129 J/kg·K

	Thermal conductivity, Ag	429 W/m·K
	Density, Ag	10500 kg/m^3
	Heat capacity, Ag	235 J/kg·K
	Thermal conductivity, Parylene-C	0.084 W/m·K
	Density, Parylene-C	1289 kg/m^3
	Heat capacity, Parylene-C	712 J/kg·K
	Thermal conductivity, MoS_2	23.2 W/m·K [5]
	Density, MoS_2	5060 kg/m^3
	Heat capacity, MoS_2	87.5 J/kg·K (14 J/mol·K) [6]
**Thermal Expansion (Multiphysics)**	Thermal expansion coefficient, Au	1.42x10^{-5} /K
	Thermal expansion coefficient, Ag	1.89x10^{-5} /K
	Thermal expansion coefficient, Parylene-C	3.5x10^{-5} /K
	Thermal expansion coefficient, MoS_2	7.6x10^{-6} /K [7]

Supplementary Figures

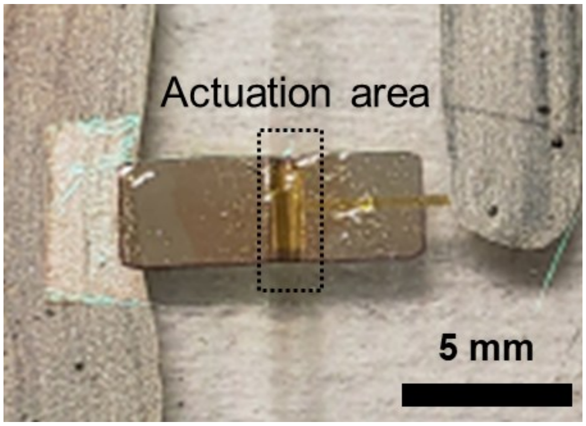

Figure S1. A photo of monolayer MoS_2 flexo-actuator.

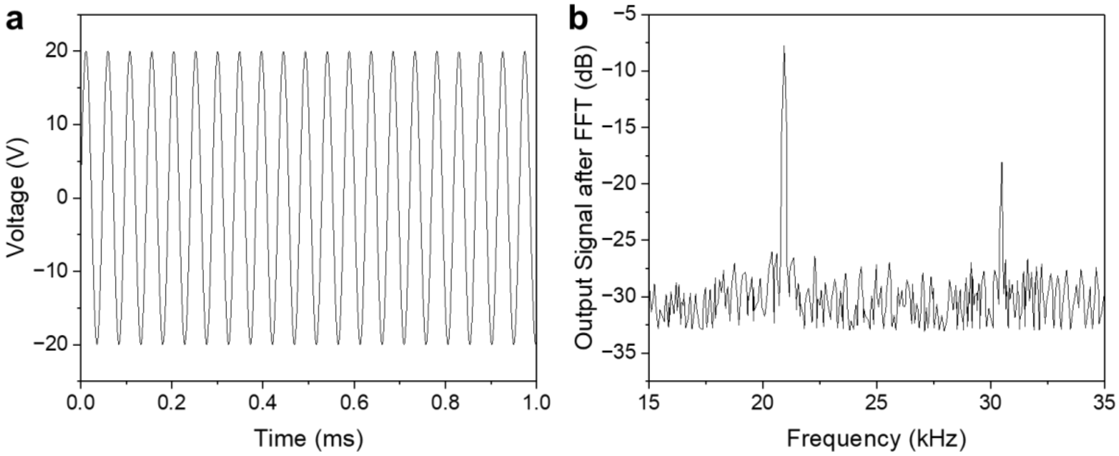

Figure S2. (a) AC voltage applied to the device at the 5th harmonic frequency for actuation. (b) Output voltage signal from the laser vibrometer after fast Fourier transform (FFT), which is converted to vertical velocity and then to displacement.

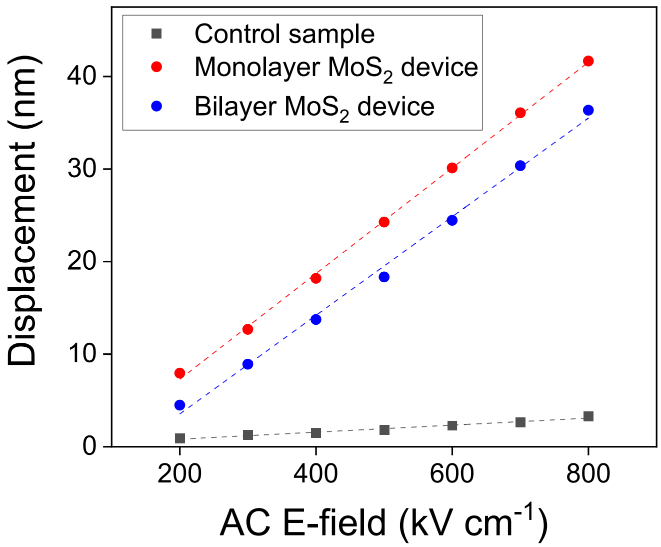

Figure S3. Displacement of three different types of devices (control, monolayer MoS_2, and bilayer MoS_2 devices) at resonant frequency under different AC electric-field (E-field). AC E-field was controlled by varying peak-to-peak voltage.

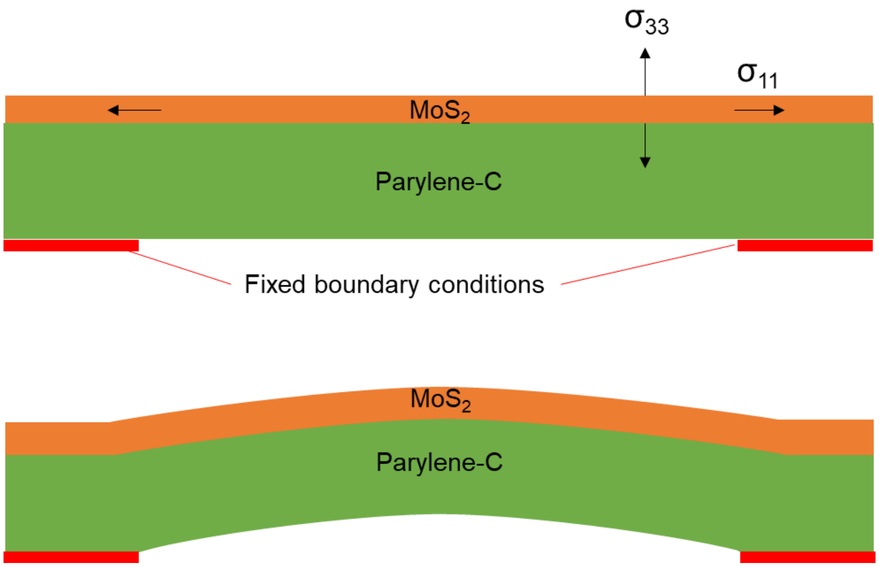

Figure S4. Schematic diagram of beam bending induced by in-plane stress of MoS_2 layer.

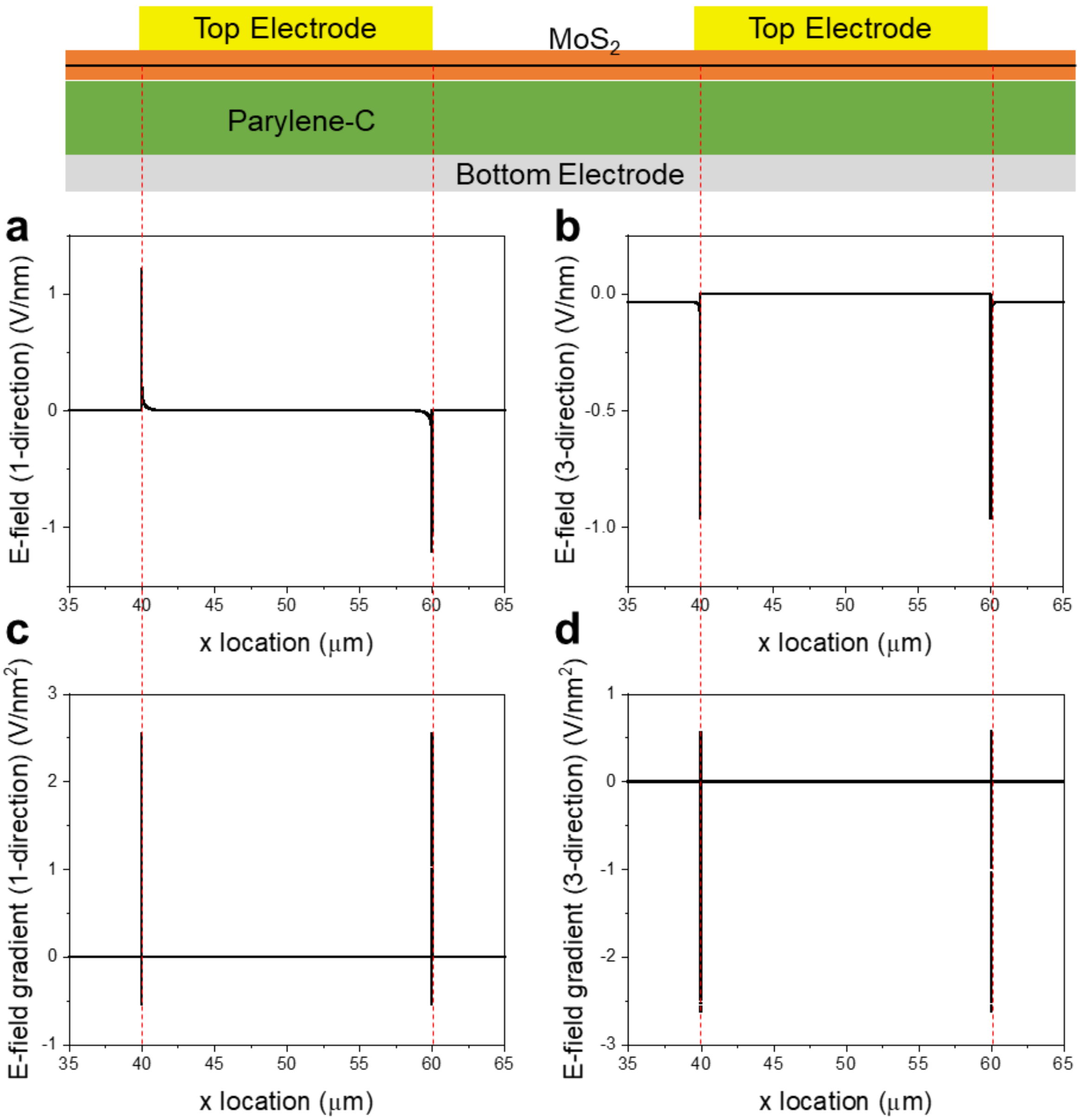

Figure S5. E-field (a) E_1 and (b) E_3, and electric-field (E-field) gradient (c) $\partial E_1/\partial x_1$ and (d) $\partial E_3/\partial x_3$ values at mid-plane of MoS_2 layer via electromagnetic simulation.

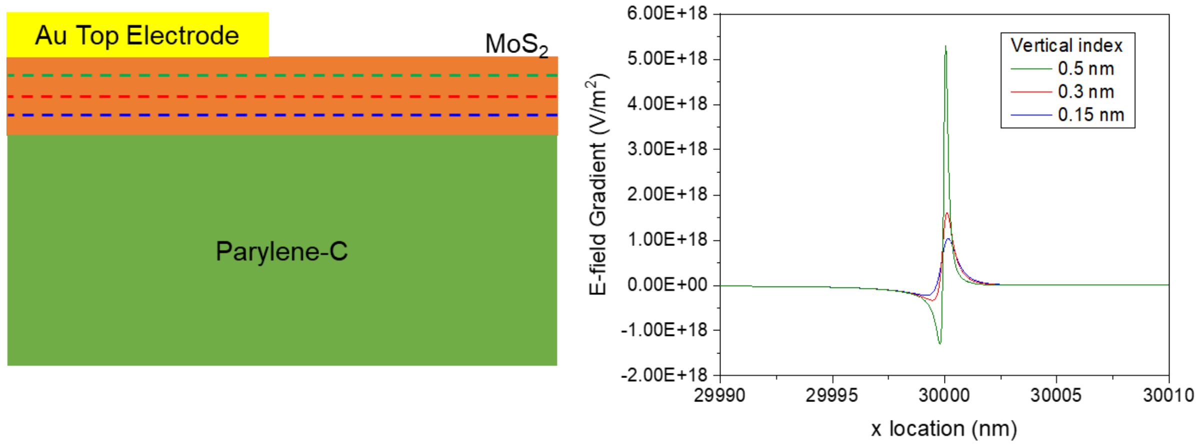

Figure S6. Electric-field (E-field) gradient $\partial E_1/\partial x_1$ value at different locations of MoS_2 layer via COMSOL electromagnetic simulation.

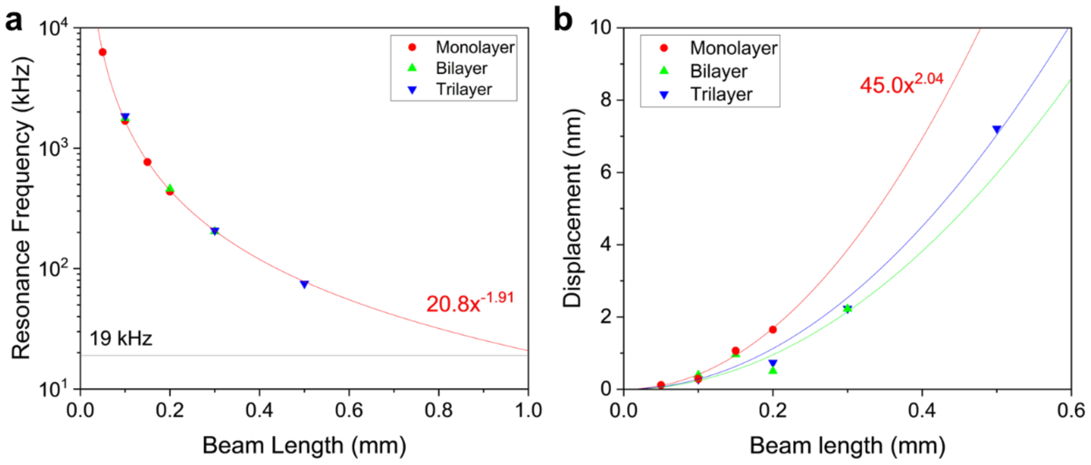

Figure S7. (a) Resonant frequency trends for actuators with different MoS_2 layer thicknesses. A quadratically decreasing trend is maintained as the MoS_2 layer becomes thicker with increasing layer number. (b) Fifth-harmonic displacement for different MoS_2 layer thicknesses. Although the trend is maintained, the bi- and tri-layer MoS_2 actuators exhibit lower displacement compared to the monolayer MoS_2 actuator.

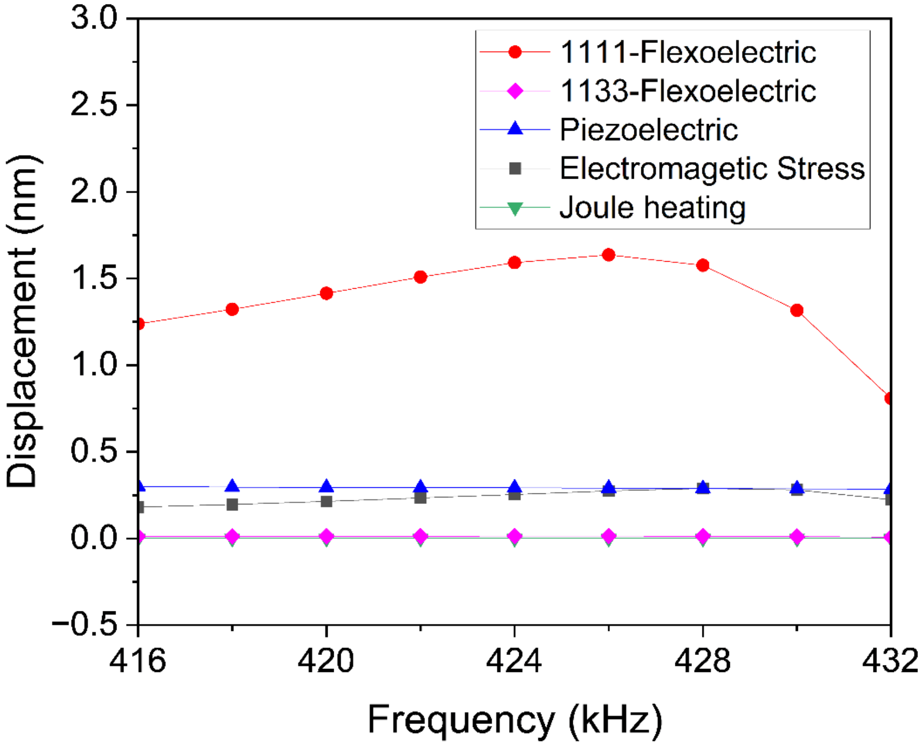

Figure S8. Fifth-harmonic displacement resulting from the 1111- and 1133-converse flexoelectric effects, piezoelectric effect, electromagnetic stress, and Joule heating. A 200 μm-long model with monolayer MoS_2 was implemented for the calculations, for which the 5th resonant frequency is estimated to be ~430 kHz.

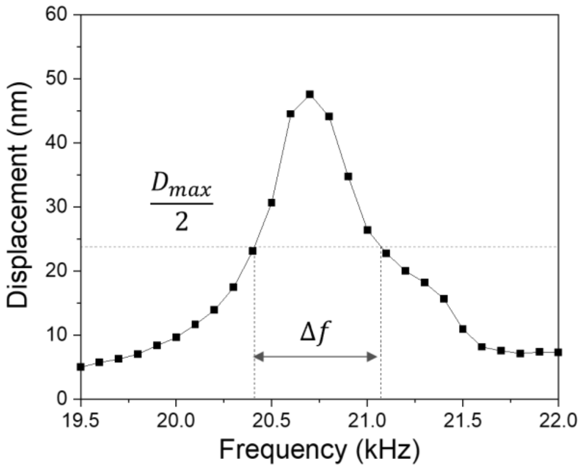

Figure S9. Fifth-harmonic displacement of the monolayer MoS_2 actuator near the resonant frequency range for Q-factor calcuation. D_{max} and Δf indicate the maximum displacement and the width of the peak at its half height.

Supplementary References

1. Peng, Q. & De, S. Outstanding mechanical properties of monolayer MoS_2 and its application in elastic energy storage. *Phys Chem Chem Phys* **15**, 19427-19437 (2013).

2. Yamusa, S. A. *et al.* Elucidating the Structural, Electronic, Elastic, and Optical Properties of Bulk and Monolayer MoS_2 Transition-Metal Dichalcogenides: A DFT Approach. *ACS Omega* **7**, 45719-45731 (2022).

3. Zhu, H. *et al.* Observation of piezoelectricity in free-standing monolayer MoS_2. *Nat Nanotechnol* **10**, 151-155 (2014).

4. Santos, E. J. G. & Kaxiras, E. Electrically Driven Tuning of the Dielectric Constant in MoS_2 Layers. *ACS Nano* **7**, 10741-10746 (2013).

5. Cai, Y., Lan, J., Zhang, G. & Zhang, Y. W. Lattice vibrational modes and phonon thermal conductivity of monolayer MoS_2. *Phys Rev B* **89**, 035438 (2014).

6. Xue, L. *et al.* The mechanical and thermal parameters of two-dimensional hexagonal materials evaluated using elastic properties: Monolayer MoS_2 as an example. *Results Phys* **57**, 107418 (2024).

7. Zhang, L. *et al.* Thermal Expansion Coefficient of Monolayer Molybdenum Disulfide Using Micro-Raman Spectroscopy. *Nano Lett* **19**, 4745-4751 (2019).
